# Pyrrolidin-1-ium 2-(naphthalen-1-yl)acetate–2-(naphthalen-1-yl)acetic acid (1/1)

**DOI:** 10.1107/S160053681201001X

**Published:** 2012-03-17

**Authors:** Zhao Hong, Fu-Jun Yin, Xing-You Xu, Li-Jun Han, Li Ren

**Affiliations:** a Department of Chemical Engineering, Huaihai Institute of Technology, Lianyungang 222005, People’s Republic of China; b Jiangsu Marine Resources Development Research Institute, Huaihai Institute of Technology, Lianyungang 222005, People’s Republic of China; c Huaiyin Insititute of Technology, Huaiyin 223003, People’s Republic of China; d Department of Mathematics and Science, Huaihai Institute of Technology, Lianyungang 222005, People’s Republic of China; e Qian’an College, Hebei United University, Tangshan 063009, People’s Republic of China

## Abstract

In the title compound, C_4_H_10_N^+^·C_12_H_9_O_2_^−^·C_12_H_10_O_2_, the pyrrolidine ring adopts an envelope conformation and the dihedral angle between the planes of the two naphthalene ring systems is 8.34 (10)°. The crystal structure is stabilized by O—H⋯O and N—H⋯O hydrogen bonds.

## Related literature

For the crystal structures of related naphthalene-1-yl-acetate complexes, see: Yin *et al.* (2010 ▶); Chen *et al.* (2004 ▶); Yang *et al.* (2008 ▶); Tang *et al.* (2006 ▶); Ji *et al.* (2011 ▶). 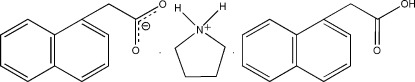


## Experimental

### 

#### Crystal data

C_4_H_10_N^+^·C_12_H_9_O_2_^−^·C_12_H_10_O_2_
*M* *_r_* = 443.52
Monoclinic, 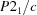

*a* = 9.4696 (12) Å
*b* = 19.359 (2) Å
*c* = 14.3888 (14) Å
β = 115.975 (6)°
*V* = 2371.3 (5) Å^3^
*Z* = 4
Mo *K*α radiation
μ = 0.08 mm^−1^
*T* = 298 K
0.24 × 0.18 × 0.15 mm

#### Data collection

Bruker APEXII CCD area-detector diffractometer
Absorption correction: multi-scan (*SADABS*; Bruker, 2008) *T*_min_ = 0.980, *T*_max_ = 0.988
21508 measured reflections
5453 independent reflections
3296 reflections with *I* > 2σ(*I*)
*R*_int_ = 0.024

#### Refinement

*R*[*F* ^2^ > 2σ(*F* ^2^)] = 0.062
*wR*(*F* ^2^) = 0.211
*S* = 1.05
5453 reflections
298 parameters
1 restraint
H-atom parameters constrained
Δρ_max_ = 0.43 e Å^−3^
Δρ_min_ = −0.22 e Å^−3^

### 

Data collection: *APEX2* (Bruker, 2008 ▶); cell refinement: *SAINT* (Bruker, 2008 ▶); data reduction: *SAINT*; program(s) used to solve structure: *SHELXS97* (Sheldrick, 2008 ▶); program(s) used to refine structure: *SHELXL97* (Sheldrick, 2008 ▶); molecular graphics: *DIAMOND* (Brandenburg & Berndt, 1999 ▶); software used to prepare material for publication: *SHELXL97*.

## Supplementary Material

Crystal structure: contains datablock(s) I, global. DOI: 10.1107/S160053681201001X/wn2468sup1.cif

Structure factors: contains datablock(s) I. DOI: 10.1107/S160053681201001X/wn2468Isup2.hkl

Supplementary material file. DOI: 10.1107/S160053681201001X/wn2468Isup3.cml

Additional supplementary materials:  crystallographic information; 3D view; checkCIF report

## Figures and Tables

**Table 1:** Hydrogen-bond geometry (Å, °)

*D*—H⋯*A*	*D*—H	H⋯*A*	*D*⋯*A*	*D*—H⋯*A*
O1*B*—H1*B*⋯O2*A*	0.82	1.77	2.581 (2)	170
N1*C*—H1*C*3⋯O2*A*	0.90	1.83	2.728 (3)	175
N1*C*—H1*C*4⋯O1*A*^i^	0.90	1.83	2.719 (3)	169

Symmetry code: (i) 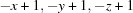.

**Fig. 1.**
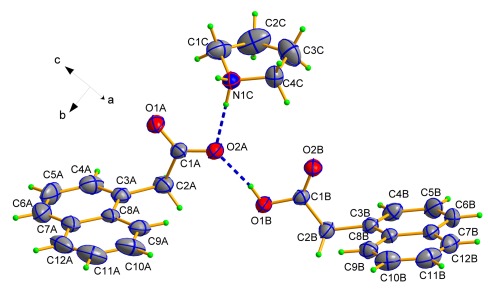
The structure of the asymmetric unit of the title compound. Displacement ellipsoids are drawn at the 30% probability level. Hydrogen bonding is shown as dashed lines.

**Fig. 2.**
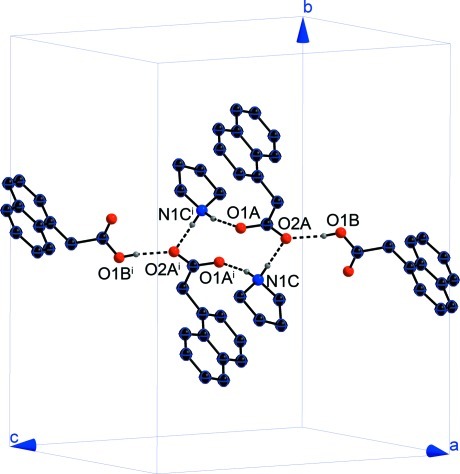
Part of the crystal structure of the title compound. Hydrogen bonding is shown as dashed lines and H atoms not involved in hydrogen bonding are omitted for clarity [Symmetry code: (i) -x+1, -y+1, -z+1].

## supplementary crystallographic information

### Comment

1-Naphthyl acetate is well known as a ligand capable of forming transition metal complexes (Yin **et al.**, 2011; Liu *et al.*, 2007; Yang *et al.*, 2008; Tang *et al.*, 2006 ; Ji *et al.*, 2011). We intended to prepare a copper(II) complex of 1-naphthyl acetate and the co-ligand pyrrolidine, but the title compound was obtained and we report its crystal strcture here.

The pyrrolidine ring adopts an envelope conformation, with C1C as the flap atom, and the dihedral angle between the planes of the two naphthalene ring systems is 8.34 (10)° (Fig. 1). The crystal structure is stabilized by intermolecular O—H···O and N—H···O hydrogen bond interactions (Fig. 2 and Table 1).

### Experimental

The title compound was synthesized by the reaction of 1-naphthylacetic acid (93 mg, 0.5 mmol), pyrrolidine (17.78 mg, 0.25 mmol) and cupric acetate (100 mg, 0.5 mmol), in 16 ml of a water-ethanol (2:1) mixture under solvothermal conditions. The mixture was homogenized and transferred to a sealed Teflon-lined solvothermal bomb (volume 25 ml) and heated to 120°C for three days. After cooling, colorless crystals of the title compound were obtained.

### Refinement

All H atoms were positioned geometrically and refined using a riding model with Csp^2^—H = 0.93 Å, Cmethylene—H = 0.97 Å; O—H = 0.82 Å and N—H = 0.90 Å; *U*_iso_(H) = x*U*_eq_(C,N,O), where x = 1.5 for O—H and 1.2 for all other H atoms.

### Crystal data

**Table:**

C_4_H_10_N^+^·C_12_H_9_O_2_^−^·C_12_H_10_O_2_	*F*(000) = 944
*M**_r_* = 443.52	*D*_x_ = 1.242 Mg m^−^^3^
Monoclinic, *P*2_1_/*c*	Mo *K*α radiation, λ = 0.71073 Å
Hall symbol: -P 2ybc	Cell parameters from 2134 reflections
*a* = 9.4696 (12) Å	θ = 2.6–26.3°
*b* = 19.359 (2) Å	µ = 0.08 mm^−^^1^
*c* = 14.3888 (14) Å	*T* = 298 K
β = 115.975 (6)°	Block, colourless
*V* = 2371.3 (5) Å^3^	0.24 × 0.18 × 0.15 mm
*Z* = 4	

### Data collection

**Table:**

Bruker APEXII CCD area-detector diffractometer	5453 independent reflections
Radiation source: fine-focus sealed tube	3296 reflections with *I* > 2σ(*I*)
Graphite monochromator	*R*_int_ = 0.024
φ and ω scans	θ_max_ = 27.5°, θ_min_ = 2.4°
Absorption correction: multi-scan (*SADABS*; Bruker, 2008)	*h* = −12→12
*T*_min_ = 0.980, *T*_max_ = 0.988	*k* = −25→25
21508 measured reflections	*l* = −18→18

### Refinement

**Table:**

Refinement on *F*^2^	Primary atom site location: structure-invariant direct methods
Least-squares matrix: full	Secondary atom site location: difference Fourier map
*R*[*F*^2^ > 2σ(*F*^2^)] = 0.062	Hydrogen site location: inferred from neighbouring sites
*wR*(*F*^2^) = 0.211	H-atom parameters constrained
*S* = 1.05	*w* = 1/[σ^2^(*F*_o_^2^) + (0.1035*P*)^2^ + 0.5472*P*] where *P* = (*F*_o_^2^ + 2*F*_c_^2^)/3
5453 reflections	(Δ/σ)_max_ < 0.001
298 parameters	Δρ_max_ = 0.43 e Å^−^^3^
1 restraint	Δρ_min_ = −0.22 e Å^−^^3^

### Special details

**Table:**

Geometry. All esds (except the esd in the dihedral angle between two l.s. planes) are estimated using the full covariance matrix. The cell esds are taken into account individually in the estimation of esds in distances, angles and torsion angles; correlations between esds in cell parameters are only used when they are defined by crystal symmetry. An approximate (isotropic) treatment of cell esds is used for estimating esds involving l.s. planes.
Refinement. Refinement of F^2^ against ALL reflections. The weighted R-factor wR and goodness of fit S are based on F^2^, conventional R-factors R are based on F, with F set to zero for negative F^2^. The threshold expression of F^2^ > 2sigma(F^2^) is used only for calculating R-factors(gt) etc. and is not relevant to the choice of reflections for refinement. R-factors based on F^2^ are statistically about twice as large as those based on F, and R- factors based on ALL data will be even larger.

### Fractional atomic coordinates and isotropic or equivalent isotropic displacement parameters (Å^2^)

**Table:**

	*x*	*y*	*z*	*U*_iso_*/*U*_eq_	
C1A	0.7694 (3)	0.56919 (12)	0.50959 (19)	0.0600 (6)	
C2A	0.8778 (3)	0.63106 (13)	0.5294 (2)	0.0737 (7)	
H2A1	0.9837	0.6173	0.5766	0.088*	
H2A2	0.8789	0.6445	0.4648	0.088*	
C3A	0.8323 (3)	0.69269 (12)	0.57397 (19)	0.0647 (6)	
C4A	0.9099 (4)	0.70977 (17)	0.6750 (2)	0.0933 (9)	
H4A	0.9963	0.6836	0.7179	0.112*	
C5A	0.8630 (6)	0.7670 (2)	0.7178 (3)	0.1164 (13)	
H5A	0.9199	0.7786	0.7871	0.140*	
C6A	0.7344 (6)	0.80433 (19)	0.6562 (3)	0.1088 (11)	
H6A	0.7020	0.8407	0.6844	0.131*	
C7A	0.6519 (4)	0.78932 (13)	0.5536 (2)	0.0806 (8)	
C8A	0.7010 (3)	0.73361 (11)	0.51151 (19)	0.0606 (6)	
C9A	0.6117 (3)	0.71985 (14)	0.4042 (2)	0.0791 (7)	
H9A	0.6416	0.6832	0.3749	0.095*	
C10A	0.4867 (5)	0.75741 (19)	0.3438 (3)	0.1127 (13)	
H10A	0.4324	0.7471	0.2739	0.135*	
C11A	0.4388 (5)	0.8109 (2)	0.3846 (4)	0.1167 (13)	
H11A	0.3505	0.8360	0.3417	0.140*	
C12A	0.5152 (4)	0.82859 (16)	0.4856 (4)	0.1041 (11)	
H12A	0.4803	0.8657	0.5111	0.125*	
O1A	0.7109 (2)	0.55882 (9)	0.56956 (15)	0.0790 (5)	
O2A	0.7498 (2)	0.52969 (9)	0.43444 (13)	0.0746 (5)	
C1B	0.8789 (2)	0.49657 (11)	0.25477 (15)	0.0510 (5)	
C2B	0.9675 (3)	0.51230 (13)	0.19270 (18)	0.0632 (6)	
H2B1	1.0765	0.5205	0.2403	0.076*	
H2B2	0.9262	0.5549	0.1549	0.076*	
C3B	0.9623 (2)	0.45772 (11)	0.11699 (16)	0.0544 (5)	
C4B	1.0854 (3)	0.41337 (14)	0.14395 (19)	0.0705 (6)	
H4B	1.1685	0.4166	0.2096	0.085*	
C5B	1.0904 (3)	0.36336 (15)	0.0760 (2)	0.0816 (8)	
H5B	1.1762	0.3337	0.0970	0.098*	
C6B	0.9730 (3)	0.35751 (13)	−0.0191 (2)	0.0759 (7)	
H6B	0.9780	0.3238	−0.0637	0.091*	
C7B	0.8406 (3)	0.40219 (12)	−0.05298 (17)	0.0606 (6)	
C8B	0.8342 (2)	0.45270 (10)	0.01616 (16)	0.0507 (5)	
C9B	0.7024 (3)	0.49680 (12)	−0.0182 (2)	0.0654 (6)	
H9B	0.6957	0.5298	0.0268	0.078*	
C10B	0.5854 (3)	0.49204 (17)	−0.1154 (2)	0.0854 (8)	
H10B	0.4994	0.5215	−0.1362	0.102*	
C11B	0.5930 (4)	0.4435 (2)	−0.1842 (2)	0.0950 (10)	
H11B	0.5133	0.4413	−0.2513	0.114*	
C12B	0.7175 (4)	0.39886 (16)	−0.1536 (2)	0.0817 (8)	
H12B	0.7208	0.3660	−0.1998	0.098*	
O1B	0.8904 (2)	0.54736 (8)	0.31785 (13)	0.0770 (5)	
H1B	0.8408	0.5377	0.3506	0.115*	
O2B	0.80792 (19)	0.44414 (8)	0.24999 (13)	0.0694 (5)	
C1C	0.6675 (5)	0.3887 (2)	0.5480 (2)	0.1064 (11)	
H1C1	0.7275	0.4240	0.5973	0.128*	
H1C2	0.5964	0.3670	0.5713	0.128*	
C2C	0.7708 (4)	0.3378 (3)	0.5353 (4)	0.1417 (17)	
H2C1	0.7846	0.2987	0.5806	0.170*	
H2C2	0.8730	0.3580	0.5527	0.170*	
C3C	0.6962 (4)	0.3147 (2)	0.4238 (4)	0.1201 (13)	
H3C1	0.7734	0.3138	0.3965	0.144*	
H3C2	0.6521	0.2687	0.4178	0.144*	
C4C	0.5702 (4)	0.36571 (15)	0.3665 (2)	0.0844 (8)	
H4C1	0.5872	0.3868	0.3111	0.101*	
H4C2	0.4679	0.3437	0.3373	0.101*	
N1C	0.5812 (2)	0.41811 (10)	0.44469 (15)	0.0695 (5)	
H1C3	0.6313	0.4559	0.4380	0.083*	
H1C4	0.4842	0.4307	0.4351	0.083*	

### Atomic displacement parameters (Å^2^)

**Table:**

	*U*^11^	*U*^22^	*U*^33^	*U*^12^	*U*^13^	*U*^23^
C1A	0.0622 (12)	0.0554 (12)	0.0756 (15)	0.0001 (10)	0.0423 (12)	0.0029 (11)
C2A	0.0671 (14)	0.0686 (15)	0.1000 (19)	−0.0054 (11)	0.0500 (14)	0.0030 (13)
C3A	0.0682 (14)	0.0604 (13)	0.0689 (12)	−0.0184 (11)	0.0333 (11)	−0.0001 (11)
C4A	0.096 (2)	0.095 (2)	0.0720 (14)	−0.0309 (17)	0.0205 (15)	0.0050 (15)
C5A	0.159 (3)	0.126 (3)	0.0610 (18)	−0.062 (3)	0.046 (2)	−0.034 (2)
C6A	0.156 (3)	0.086 (2)	0.108 (3)	−0.033 (2)	0.079 (3)	−0.023 (2)
C7A	0.107 (2)	0.0526 (14)	0.109 (2)	−0.0124 (14)	0.0718 (18)	−0.0010 (14)
C8A	0.0724 (14)	0.0462 (11)	0.0762 (15)	−0.0109 (10)	0.0445 (12)	0.0049 (10)
C9A	0.0936 (18)	0.0656 (15)	0.0737 (16)	−0.0201 (14)	0.0325 (14)	0.0098 (13)
C10A	0.115 (3)	0.082 (2)	0.106 (2)	−0.023 (2)	0.015 (2)	0.034 (2)
C11A	0.106 (3)	0.081 (2)	0.146 (4)	−0.005 (2)	0.040 (3)	0.036 (2)
C12A	0.113 (2)	0.0542 (16)	0.173 (4)	0.0067 (17)	0.088 (3)	0.019 (2)
O1A	0.0956 (12)	0.0729 (11)	0.0980 (13)	−0.0223 (9)	0.0697 (11)	−0.0171 (9)
O2A	0.0976 (12)	0.0710 (10)	0.0748 (11)	−0.0163 (9)	0.0556 (10)	−0.0103 (8)
C1B	0.0507 (10)	0.0544 (12)	0.0461 (10)	−0.0010 (9)	0.0197 (9)	0.0011 (9)
C2B	0.0696 (13)	0.0701 (14)	0.0590 (12)	−0.0166 (11)	0.0366 (11)	−0.0117 (11)
C3B	0.0564 (11)	0.0597 (12)	0.0548 (12)	−0.0046 (10)	0.0315 (10)	0.0001 (9)
C4B	0.0621 (13)	0.0869 (18)	0.0621 (14)	0.0084 (12)	0.0268 (11)	0.0107 (12)
C5B	0.0839 (17)	0.0836 (18)	0.0889 (19)	0.0283 (14)	0.0484 (16)	0.0166 (15)
C6B	0.104 (2)	0.0592 (14)	0.0893 (19)	0.0052 (13)	0.0657 (17)	−0.0066 (13)
C7B	0.0759 (14)	0.0577 (13)	0.0592 (13)	−0.0113 (11)	0.0397 (11)	−0.0044 (10)
C8B	0.0564 (11)	0.0486 (11)	0.0553 (11)	−0.0034 (9)	0.0320 (10)	0.0012 (9)
C9B	0.0627 (13)	0.0634 (14)	0.0743 (15)	0.0031 (11)	0.0340 (12)	0.0071 (11)
C10B	0.0598 (14)	0.097 (2)	0.087 (2)	0.0012 (14)	0.0210 (14)	0.0242 (17)
C11B	0.0810 (19)	0.124 (3)	0.0603 (16)	−0.0340 (19)	0.0128 (14)	0.0076 (17)
C12B	0.098 (2)	0.0851 (18)	0.0629 (15)	−0.0318 (16)	0.0365 (15)	−0.0146 (13)
O1B	0.1145 (14)	0.0647 (10)	0.0784 (11)	−0.0216 (9)	0.0669 (11)	−0.0151 (8)
O2B	0.0770 (10)	0.0655 (10)	0.0799 (11)	−0.0185 (8)	0.0474 (9)	−0.0119 (8)
C1C	0.116 (3)	0.111 (3)	0.078 (2)	−0.034 (2)	0.0304 (18)	0.0048 (18)
C2C	0.078 (2)	0.205 (5)	0.127 (3)	0.014 (3)	0.032 (2)	0.053 (3)
C3C	0.095 (2)	0.097 (2)	0.163 (4)	0.0124 (19)	0.052 (2)	−0.016 (2)
C4C	0.099 (2)	0.0790 (18)	0.0844 (18)	0.0012 (15)	0.0488 (16)	−0.0135 (15)
N1C	0.0802 (12)	0.0663 (12)	0.0743 (13)	−0.0142 (10)	0.0452 (11)	−0.0079 (10)

### Geometric parameters (Å, º)

**Table:**

C1A—O1A	1.229 (3)	C4B—C5B	1.391 (4)
C1A—O2A	1.270 (3)	C4B—H4B	0.9300
C1A—C2A	1.521 (3)	C5B—C6B	1.338 (4)
C2A—C3A	1.504 (3)	C5B—H5B	0.9300
C2A—H2A1	0.9700	C6B—C7B	1.422 (4)
C2A—H2A2	0.9700	C6B—H6B	0.9300
C3A—C4A	1.352 (4)	C7B—C12B	1.408 (4)
C3A—C8A	1.415 (3)	C7B—C8B	1.415 (3)
C4A—C5A	1.430 (5)	C8B—C9B	1.411 (3)
C4A—H4A	0.9300	C9B—C10B	1.354 (4)
C5A—C6A	1.359 (5)	C9B—H9B	0.9300
C5A—H5A	0.9300	C10B—C11B	1.390 (5)
C6A—C7A	1.365 (5)	C10B—H10B	0.9300
C6A—H6A	0.9300	C11B—C12B	1.369 (5)
C7A—C8A	1.412 (4)	C11B—H11B	0.9300
C7A—C12A	1.450 (5)	C12B—H12B	0.9300
C8A—C9A	1.424 (4)	O1B—H1B	0.8200
C9A—C10A	1.335 (4)	C1C—C2C	1.455 (6)
C9A—H9A	0.9300	C1C—N1C	1.462 (4)
C10A—C11A	1.362 (6)	C1C—H1C1	0.9700
C10A—H10A	0.9300	C1C—H1C2	0.9700
C11A—C12A	1.353 (5)	C2C—C3C	1.510 (6)
C11A—H11A	0.9300	C2C—H2C1	0.9700
C12A—H12A	0.9300	C2C—H2C2	0.9700
C1B—O2B	1.203 (2)	C3C—C4C	1.489 (4)
C1B—O1B	1.310 (2)	C3C—H3C1	0.9700
C1B—C2B	1.500 (3)	C3C—H3C2	0.9700
C2B—C3B	1.503 (3)	C4C—N1C	1.484 (3)
C2B—H2B1	0.9700	C4C—H4C1	0.9700
C2B—H2B2	0.9700	C4C—H4C2	0.9700
C3B—C4B	1.360 (3)	N1C—H1C3	0.9000
C3B—C8B	1.430 (3)	N1C—H1C4	0.9000
			
O1A—C1A—O2A	123.8 (2)	C6B—C5B—H5B	119.7
O1A—C1A—C2A	118.1 (2)	C4B—C5B—H5B	119.7
O2A—C1A—C2A	118.0 (2)	C5B—C6B—C7B	120.8 (2)
C3A—C2A—C1A	114.16 (18)	C5B—C6B—H6B	119.6
C3A—C2A—H2A1	108.7	C7B—C6B—H6B	119.6
C1A—C2A—H2A1	108.7	C12B—C7B—C8B	118.9 (2)
C3A—C2A—H2A2	108.7	C12B—C7B—C6B	122.2 (2)
C1A—C2A—H2A2	108.7	C8B—C7B—C6B	118.9 (2)
H2A1—C2A—H2A2	107.6	C9B—C8B—C7B	118.4 (2)
C4A—C3A—C8A	117.3 (3)	C9B—C8B—C3B	122.8 (2)
C4A—C3A—C2A	122.0 (3)	C7B—C8B—C3B	118.79 (19)
C8A—C3A—C2A	120.6 (2)	C10B—C9B—C8B	121.2 (3)
C3A—C4A—C5A	121.8 (3)	C10B—C9B—H9B	119.4
C3A—C4A—H4A	119.1	C8B—C9B—H9B	119.4
C5A—C4A—H4A	119.1	C9B—C10B—C11B	120.5 (3)
C6A—C5A—C4A	119.4 (3)	C9B—C10B—H10B	119.7
C6A—C5A—H5A	120.3	C11B—C10B—H10B	119.7
C4A—C5A—H5A	120.3	C12B—C11B—C10B	120.2 (3)
C5A—C6A—C7A	121.1 (3)	C12B—C11B—H11B	119.9
C5A—C6A—H6A	119.4	C10B—C11B—H11B	119.9
C7A—C6A—H6A	119.4	C11B—C12B—C7B	120.7 (3)
C6A—C7A—C8A	119.1 (3)	C11B—C12B—H12B	119.7
C6A—C7A—C12A	122.5 (3)	C7B—C12B—H12B	119.7
C8A—C7A—C12A	118.4 (3)	C1B—O1B—H1B	109.5
C7A—C8A—C3A	121.2 (2)	C2C—C1C—N1C	104.1 (3)
C7A—C8A—C9A	117.2 (2)	C2C—C1C—H1C1	110.9
C3A—C8A—C9A	121.6 (2)	N1C—C1C—H1C1	110.9
C10A—C9A—C8A	122.7 (3)	C2C—C1C—H1C2	110.9
C10A—C9A—H9A	118.7	N1C—C1C—H1C2	110.9
C8A—C9A—H9A	118.7	H1C1—C1C—H1C2	109.0
C9A—C10A—C11A	120.1 (4)	C1C—C2C—C3C	107.8 (3)
C9A—C10A—H10A	120.0	C1C—C2C—H2C1	110.1
C11A—C10A—H10A	120.0	C3C—C2C—H2C1	110.1
C12A—C11A—C10A	122.2 (4)	C1C—C2C—H2C2	110.1
C12A—C11A—H11A	118.9	C3C—C2C—H2C2	110.1
C10A—C11A—H11A	118.9	H2C1—C2C—H2C2	108.5
C11A—C12A—C7A	119.4 (3)	C4C—C3C—C2C	106.3 (3)
C11A—C12A—H12A	120.3	C4C—C3C—H3C1	110.5
C7A—C12A—H12A	120.3	C2C—C3C—H3C1	110.5
O2B—C1B—O1B	123.24 (19)	C4C—C3C—H3C2	110.5
O2B—C1B—C2B	125.56 (19)	C2C—C3C—H3C2	110.5
O1B—C1B—C2B	111.19 (18)	H3C1—C3C—H3C2	108.7
C1B—C2B—C3B	116.09 (18)	N1C—C4C—C3C	105.1 (3)
C1B—C2B—H2B1	108.3	N1C—C4C—H4C1	110.7
C3B—C2B—H2B1	108.3	C3C—C4C—H4C1	110.7
C1B—C2B—H2B2	108.3	N1C—C4C—H4C2	110.7
C3B—C2B—H2B2	108.3	C3C—C4C—H4C2	110.7
H2B1—C2B—H2B2	107.4	H4C1—C4C—H4C2	108.8
C4B—C3B—C8B	119.1 (2)	C1C—N1C—C4C	109.0 (2)
C4B—C3B—C2B	119.0 (2)	C1C—N1C—H1C3	109.9
C8B—C3B—C2B	121.8 (2)	C4C—N1C—H1C3	109.9
C3B—C4B—C5B	121.9 (2)	C1C—N1C—H1C4	109.9
C3B—C4B—H4B	119.1	C4C—N1C—H1C4	109.9
C5B—C4B—H4B	119.1	H1C3—N1C—H1C4	108.3
C6B—C5B—C4B	120.6 (2)		
			
O1A—C1A—C2A—C3A	−34.4 (3)	C1B—C2B—C3B—C8B	83.3 (3)
O2A—C1A—C2A—C3A	148.4 (2)	C8B—C3B—C4B—C5B	0.2 (3)
C1A—C2A—C3A—C4A	103.6 (3)	C2B—C3B—C4B—C5B	−177.6 (2)
C1A—C2A—C3A—C8A	−73.7 (3)	C3B—C4B—C5B—C6B	0.2 (4)
C8A—C3A—C4A—C5A	−0.4 (4)	C4B—C5B—C6B—C7B	0.0 (4)
C2A—C3A—C4A—C5A	−177.8 (3)	C5B—C6B—C7B—C12B	177.9 (3)
C3A—C4A—C5A—C6A	1.8 (5)	C5B—C6B—C7B—C8B	−0.6 (4)
C4A—C5A—C6A—C7A	−1.8 (5)	C12B—C7B—C8B—C9B	1.4 (3)
C5A—C6A—C7A—C8A	0.5 (5)	C6B—C7B—C8B—C9B	180.0 (2)
C5A—C6A—C7A—C12A	−179.4 (3)	C12B—C7B—C8B—C3B	−177.58 (19)
C6A—C7A—C8A—C3A	0.9 (3)	C6B—C7B—C8B—C3B	1.0 (3)
C12A—C7A—C8A—C3A	−179.2 (2)	C4B—C3B—C8B—C9B	−179.7 (2)
C6A—C7A—C8A—C9A	−179.9 (2)	C2B—C3B—C8B—C9B	−1.9 (3)
C12A—C7A—C8A—C9A	0.0 (3)	C4B—C3B—C8B—C7B	−0.8 (3)
C4A—C3A—C8A—C7A	−1.0 (3)	C2B—C3B—C8B—C7B	176.95 (18)
C2A—C3A—C8A—C7A	176.5 (2)	C7B—C8B—C9B—C10B	−1.0 (3)
C4A—C3A—C8A—C9A	179.9 (2)	C3B—C8B—C9B—C10B	177.8 (2)
C2A—C3A—C8A—C9A	−2.6 (3)	C8B—C9B—C10B—C11B	−0.4 (4)
C7A—C8A—C9A—C10A	0.4 (4)	C9B—C10B—C11B—C12B	1.4 (4)
C3A—C8A—C9A—C10A	179.5 (2)	C10B—C11B—C12B—C7B	−1.1 (4)
C8A—C9A—C10A—C11A	−0.8 (5)	C8B—C7B—C12B—C11B	−0.3 (4)
C9A—C10A—C11A—C12A	1.0 (5)	C6B—C7B—C12B—C11B	−178.9 (2)
C10A—C11A—C12A—C7A	−0.7 (5)	N1C—C1C—C2C—C3C	−25.7 (4)
C6A—C7A—C12A—C11A	−179.9 (3)	C1C—C2C—C3C—C4C	14.4 (5)
C8A—C7A—C12A—C11A	0.2 (4)	C2C—C3C—C4C—N1C	2.8 (4)
O2B—C1B—C2B—C3B	1.4 (3)	C2C—C1C—N1C—C4C	28.0 (4)
O1B—C1B—C2B—C3B	−179.78 (19)	C3C—C4C—N1C—C1C	−19.1 (3)
C1B—C2B—C3B—C4B	−98.9 (3)		

### Hydrogen-bond geometry (Å, º)

**Table:**

*D*—H···*A*	*D*—H	H···*A*	*D*···*A*	*D*—H···*A*
O1*B*—H1*B*···O2*A*	0.82	1.77	2.581 (2)	170
N1*C*—H1*C*3···O2*A*	0.90	1.83	2.728 (3)	175
N1*C*—H1*C*4···O1*A*^i^	0.90	1.83	2.719 (3)	169

Symmetry code: (i) −*x*+1, −*y*+1, −*z*+1.
